# Predictors of Severe Hospitalization in Patients Admitted With Acute Exacerbation of Chronic Obstructive Pulmonary Disease in a Community Hospital: A Retrospective Chart Review

**DOI:** 10.7759/cureus.117762

**Published:** 2026-10-11

**Authors:** Prabin Thapa

**Affiliations:** 1 Family Medicine, Jackson Park Hospital, Chicago, USA

**Keywords:** acute exacerbation of copd, aecopd, calibration, chart review, heart failure, icu admission, length of stay, noninvasive ventilation, readmission, risk model

## Abstract

Background

Hospitalization for acute exacerbation of chronic obstructive pulmonary disease (AECOPD) is associated with substantial morbidity and healthcare utilization in community hospitals. Because in-hospital mortality may be infrequent in single-center chart reviews, composite morbidity/utilization endpoints may better capture clinically meaningful adverse hospital trajectories.

Methods

We conducted a single-center retrospective chart review at a community hospital using unique-patient index admissions only (earliest admission per medical record number (MRN)), yielding 163 patients. The primary outcome was a composite adverse hospital course defined as any ICU admission, endotracheal intubation, prolonged length of stay (LOS > 5 days; cohort median), 30-day readmission, or in-hospital mortality. Baseline comparisons used Mann-Whitney U tests and chi-square/Fisher's exact tests. Multivariable logistic regression was used for the composite outcome. Thirty-day readmission (post-discharge) occurred in 21 cases; therefore, the primary readmission analysis used a pre-specified parsimonious model (age, congestive heart failure (CHF), creatinine, and discharge against medical advice (AMA)) with Firth penalized logistic regression as a sensitivity analysis to mitigate overfitting. LOS was modeled using negative binomial regression. AUROC and calibration were assessed as internal/apparent performance (no external validation).

Results

The composite adverse outcome occurred in 105/163 (64.4%) index admissions. ICU admission occurred in 53/163 (32.5%), noninvasive ventilation (NIV) in 58/163 (35.6%), intubation in 9/163 (5.5%), in-hospital mortality in 3/163 (1.8%), and 30-day readmission in 21/163 (12.9%). In multivariable analysis of the composite outcome, CHF (adjusted odds ratio (aOR) 3.11, 95% CI 1.15-8.43) and hypercapnia (PaCO₂ per 10 mmHg; aOR 1.94, 95% CI 1.25-3.00) were associated with higher odds of adverse course. In the parsimonious standard logistic readmission model, CHF (aOR 4.16, 95% CI 1.54-11.20) and creatinine (per 0.5 mg/dL; aOR 1.23, 95% CI 1.05-1.45) were associated with readmission; estimates were attenuated in the Firth penalized model. In negative binomial LOS modeling, NIV (adjusted incidence rate ratio (aIRR) 1.58, 95% CI 1.03-2.42) and intubation (aIRR 2.97, 95% CI 1.36-6.49) were associated with longer LOS. Apparent discrimination was AUROC 0.7192 for the composite model and AUROC 0.722 for the parsimonious readmission model.

Conclusion

In this community hospital chart review of 163 unique AECOPD index admissions, an adverse hospital course was common and largely reflected utilization and prolonged recovery rather than mortality. CHF and hypercapnia were associated with adverse composite outcomes, and CHF and renal dysfunction were associated with 30-day readmission in parsimonious modeling. Performance metrics represent internal/apparent estimates without external validation; larger, externally validated studies are needed before clinical deployment of prediction tools.

## Introduction

Acute exacerbation of chronic obstructive pulmonary disease (AECOPD) are pivotal clinical events that accelerate symptom burden, impair quality of life, and drive a substantial proportion of chronic obstructive pulmonary disease (COPD)-related emergency visits and hospitalizations [1,2]. In community hospitals, AECOPD admissions represent a frequent, high-impact workload characterized by time-sensitive triage decisions (floor vs. intensive care unit (ICU)), rapid implementation of evidence-based therapy, and discharge planning aimed at preventing relapse and early readmission [3]. Despite their prevalence, single-center datasets assembled via retrospective chart review often contain few in-hospital deaths, limiting mortality-focused modeling and motivating broader, utilization-centered outcomes (e.g., ICU use, ventilatory escalation, prolonged length of stay (LOS), and 30-day readmission) that are clinically meaningful and more statistically tractable [4,5].

Contemporary guidelines emphasize that inpatient AECOPD management should include assessment of exacerbation severity and triggers, bronchodilator optimization, short-course systemic corticosteroids, antibiotics when indicated, controlled oxygen therapy, and escalation to ventilatory support for acute hypercapnic respiratory failure [3]. For patients with AECOPD accompanied by respiratory acidosis, noninvasive ventilation (NIV) is strongly supported by randomized evidence and clinical practice guidelines. The Official European Respiratory Society/American Thoracic Society (ERS/ATS) guideline recommends bilevel NIV for AECOPD with acute (or acute-on-chronic) hypercapnic respiratory failure and respiratory acidosis, reflecting high-certainty evidence that NIV reduces the need for endotracheal intubation and improves short-term outcomes compared with usual care alone [4]. Foundational randomized trials and subsequent meta-analyses established NIV as a standard of care in appropriately selected patients [6-8], and ongoing randomized work continues to refine NIV intensity strategies to reduce intubation requirements in hypercapnic AECOPD [9]. Nevertheless, a subset of patients progresses to invasive mechanical ventilation (IMV), and critical care studies consistently demonstrate that IMV and NIV failure are associated with worse outcomes and prolonged hospitalization, underscoring the relevance of escalation markers in hospital-based datasets [10-12].

Beyond acute respiratory management, multimorbidity is central to COPD outcomes, particularly in hospitalized patients. Cardiovascular disease, including congestive heart failure (CHF), commonly coexists with COPD and can complicate dyspnea evaluation, volume management, and recovery trajectory [3]. Readmission following AECOPD hospitalization is likewise a persistent clinical and health system challenge; systematic reviews and meta-analyses demonstrate that early readmission risk reflects a combination of comorbidity burden, prior utilization, severity during the index hospitalization (including LOS and ICU-level care), and discharge-related factors [5,13,14]. In this context, community hospital chart review data offer an opportunity to identify locally relevant risk signals using routinely available variables (e.g., arterial blood gas (ABG) values, basic labs, and comorbidity documentation), while acknowledging the limits of causal inference in observational designs.

Accordingly, we conducted a retrospective chart review of unique patients hospitalized for AECOPD in a community hospital, using the earliest (index) admission per patient to (1) assess predictors of composite adverse hospital course (ICU admission, intubation, prolonged LOS, 30‑day readmission, or mortality), (2) evaluate the role of comorbidities (e.g., CHF, CKD, diabetes, CAD) in influencing outcomes, (3) examine physiologic severity markers (ABG pH, PaCO₂, WBC, creatinine, BNP) as risk signals, and (4) analyze healthcare utilization outcomes, including LOS and readmission patterns.

## Materials and methods

Study design and setting

This investigation was a retrospective chart review of adult hospital admissions for AECOPD at Jackson Park Hospital in Chicago, IL, from January 2025 through December 2025. The analytic unit was the unique patient index admission to avoid non-independence associated with repeat hospitalizations. The index admission was defined as the earliest eligible hospitalization during the study period, with subsequent repeat admissions excluded. Inclusion criteria were an age of ≥18 years and admission with a primary diagnosis of AECOPD. Exclusion criteria included admissions for other primary pulmonary diagnoses (e.g., pneumonia without COPD) and incomplete medical records. The objective of this study was to describe index admission characteristics and outcomes and to evaluate associations between routinely available clinical variables and adverse hospital course and 30-day readmission. This observational study was designed for association and hypothesis generation, and no causal inferences were intended.

Data source and chart abstraction

Data were abstracted from the electronic health record (EHR) into a standardized spreadsheet using a predefined data dictionary (available upon request). Demographics and comorbidities were obtained from the problem list and admission history and physical; initial vital signs from emergency department triage; and laboratory and ABG values from the first measurements obtained during the index admission. Chest radiograph findings were classified by the presence or absence of an infiltrate based on the radiology report. In-hospital treatments and escalation of care were identified from the medication administration record and respiratory therapy and ICU documentation. Discharge outcomes and subsequent readmissions were obtained from discharge documentation and EHR readmission records.

Admission variables were recorded as the first documented value during the index admission evaluation (when multiple values were present, the earliest timestamped value was used). In-hospital variables (steroids/antibiotics, ICU admission, NIV, and intubation) were coded as ever occurred during the index hospitalization (yes/no). LOS was taken from the chart-abstracted LOS field in days.

Variables

Baseline and admission characteristics included demographic variables; comorbidities; admission vital signs; laboratory values, including ABG measurements; and the presence or absence of infiltrates on chest radiography. In-hospital course variables included LOS, systemic corticosteroid administration, and antibiotic administration. Demographic variables included age, sex, race, insurance type, smoking status, and documented illicit drug use. Comorbidities included CHF, chronic kidney disease (CKD), diabetes mellitus, and coronary artery disease (CAD). Admission physiologic variables included temperature, heart rate, respiratory rate, and oxygen saturation. Laboratory variables included ABG pH, partial pressure of carbon dioxide (PaCO₂), white blood cell (WBC) count, brain natriuretic peptide (BNP) level, and serum creatinine. Imaging variables included the presence or absence of infiltrates on chest radiography. Treatment and disease-escalation variables included systemic corticosteroid and antibiotic use, NIV, endotracheal intubation, and ICU admission. Clinical outcomes included LOS, discharge disposition, in-hospital mortality, and 30-day readmission. Missingness was assessed for all predictors used. All models were fit using complete case data for the included predictors; model-specific N is reported with results.

Outcome definitions

The primary outcome was a composite adverse hospital course, defined as the occurrence of any of the following during or after the index admission: ICU admission, endotracheal intubation, prolonged LOS (>5 days, based on the cohort median), 30-day readmission, or in-hospital mortality. Secondary outcomes included 30-day readmission and LOS (days). In-hospital mortality was reported descriptively because of its low event frequency.

Statistical analysis

All statistical analyses were performed using the index admission for each unique patient unless otherwise specified. Continuous variables were summarized as medians with interquartile ranges (IQRs), while categorical variables were presented as frequencies and percentages. For comparisons between patients with and without the composite adverse outcome, continuous variables were compared using the Mann-Whitney U test. Categorical variables were compared using the chi-square test when expected cell counts were adequate and Fisher’s exact test when they were insufficient. Test statistics were reported as U for the Mann-Whitney U test and χ² for the chi-square test. For Fisher’s exact test, the test statistic was reported as NA.

Because in-hospital mortality events were uncommon in this cohort, a multivariable model specifically for mortality was not performed. Instead, multivariable analyses focused on clinically relevant morbidity and healthcare utilization outcomes. Logistic regression was used to evaluate the composite adverse outcome and 30-day readmission. Negative binomial regression was used to model LOS because of the observed right-skewed distribution of this outcome. Adjusted associations were reported as odds ratios (ORs) or incidence rate ratios (IRRs), as appropriate, with corresponding 95% confidence intervals (CIs). Treatment and escalation variables were interpreted primarily as markers of disease severity and healthcare utilization rather than causal exposures, given the potential for confounding by indication inherent in retrospective observational data. IBM SPSS Statistics for Windows, Version 30 (Released 2024; IBM Corp; Armonk, New York, United States) was used to analyze the patient's data.

Thirty-day readmission was defined as a documented readmission within 30 days of discharge from the index admission. Because only 21 readmission events occurred, we pre-specified a parsimonious multivariable model to reduce overfitting risk and to retain clinically interpretable predictors available prior to discharge. The primary readmission model included age (years), CHF (yes/no), creatinine (per 0.5 mg/dL increase; reference range 0.70-1.30), and discharge against medical advice (AMA; yes/no). We fit this model using (1) standard logistic regression and (2) Firth penalized logistic regression, which was used as the primary inferential approach to reduce small-sample bias and separation risk. Adjusted odds ratios (aORs) with 95% CIs are reported.

For the logistic regression models evaluating the composite adverse outcome, model discrimination was assessed using the area under the receiver operating characteristic curve (AUROC), with 95% CIs estimated using bootstrap resampling. Model calibration was evaluated using decile calibration plots comparing the mean predicted probability with the observed event rate across probability deciles. A 45-degree reference line (y=x) was included to represent perfect calibration. Discrimination (AUROC) and calibration (decile calibration plots) were evaluated internally using the same dataset used for model fitting; therefore, these metrics represent apparent performance.

## Results

A total of 163 unique patients were included by restricting the dataset to each patient’s earliest (index) hospitalization (earliest admission date per medical record number (MRN)), thereby avoiding non-independence from repeat admissions. The primary endpoint (composite adverse outcome) occurred in 105/163 (64.4%) index admissions; 58/163 (35.6%) had no composite adverse outcome.

Baseline characteristics and in-hospital course by composite adverse outcome

Age and sex distribution were similar between groups. Median age was 65 years overall and did not differ by composite adverse status. Sex was balanced (approximately half male) with no significant between-group difference. Race and insurance were predominantly Black and insured, respectively; due to small counts in non-Black race categories, race was primarily interpreted descriptively. Smoking status distribution was also similar across groups. Illicit drug use prevalence did not differ significantly between composite groups.

Among the evaluated comorbidities, CHF was significantly more common in patients with a composite adverse outcome (36/105 [34.6%]) compared with those without (10/58 [17.2%]). CKD, diabetes mellitus, and CAD did not show statistically significant differences between groups in unadjusted comparisons. 

Most admission vital signs and laboratory parameters were similar between groups. Respiratory rate differed modestly, with the composite adverse group having a slightly lower median respiratory rate compared with the non-composite group. BNP showed the most prominent laboratory separation: BNP levels were substantially higher in the composite adverse group than in the non-composite group, consistent with greater cardiopulmonary stress or comorbidity burden among patients experiencing an adverse hospital course. ABG pH, PaCO₂, WBC (reported as 10E3/µL), oxygen saturation, and creatinine (mg/dL) did not show statistically significant group differences in these baseline comparisons. Chest X-ray infiltrate frequency did not differ significantly between composite groups.

LOS was longer in the composite adverse group (median 6 days) compared with the non-composite group (median 4 days), consistent with the composite definition including prolonged LOS and reflecting a more resource-intensive hospitalization overall. Antibiotic administration was also more frequent in the composite adverse group, while systemic steroid use did not differ significantly between groups. Baseline (index admission) demographics, comorbidities, admission vital signs/labs, and selected in-hospital course variables stratified by the composite adverse outcome are shown in Table 1.

**Table 1:** Baseline (index admission) and in-hospital course characteristics among unique patients with AECOPD (total N=163), stratified by composite adverse outcome. Continuous variables are presented as median [IQR] and compared using the Mann–Whitney U test (test statistic reported as U). Categorical variables are presented as n (%) and compared using the chi-square test (test statistic reported as χ²) when expected cell counts were adequate; otherwise, Fisher’s exact test was used (test statistic reported as NA) and provided P-value in the "P-value" column. AECOPD: acute exacerbation of chronic obstructive pulmonary disease; ABG: arterial blood gas; BNP: brain natriuretic peptide; CAD: coronary artery disease; CHF: congestive heart failure; CKD: chronic kidney disease; COPD: chronic obstructive pulmonary disease; IQR: interquartile range; LOS: length of stay; WBC: white blood cell.​​​​ ^*^Variables marked with an asterisk are in-hospital course variables (not baseline): LOS, steroid given, and antibiotics given reflect events/therapies during the index hospitalization. Composite adverse outcome definition: any of ICU admission, endotracheal intubation, prolonged LOS (>5 days), 30-day readmission, or in-hospital mortality.

Variable	Overall (N=163)	Composite adverse outcome: No (n=58)	Composite adverse outcome: Yes (n=105)	Test statistic	P-value
Age (years)	65.00 [58.00, 70.00]	64.00 [59.00, 68.00]	65.00 [57.00, 71.00]	U=3022	0.939
Temperature (°F)	97.90 [97.30, 98.20]	97.95 [97.33, 98.20]	97.90 [97.30, 98.20]	U=3197	0.598
Heart rate (bpm)	92.00 [80.50, 106.50]	97.50 [83.25, 110.00]	91.00 [80.00, 104.00]	U=3364	0.269
Respiratory rate (breaths/min)	20.00 [18.00, 22.50]	20.00 [18.00, 24.00]	20.00 [18.00, 22.00]	U=3673	0.0265
Oxygen saturation (%)	96.00 [93.00, 98.00]	95.50 [94.00, 97.00]	96.00 [92.00, 98.00]	U=2961	0.771
ABG pH	7.39 [7.34, 7.43]	7.38 [7.34, 7.41]	7.39 [7.34, 7.44]	U=2753	0.312
ABG PaCO₂ (mmHg)	43.60 [39.55, 53.30]	43.30 [38.42, 47.88]	44.00 [39.90, 55.00]	U=2607	0.130
WBC (×10⁹/L)	8.50 [6.35, 11.55]	8.20 [6.20, 10.55]	8.60 [6.50, 12.40]	U=2604	0.127
Creatinine (mg/dL)	0.98 [0.75, 1.37]	0.97 [0.83, 1.47]	0.98 [0.70, 1.34]	U=3305	0.368
BNP (pg/mL)	90.50 [37.00, 323.00]	45.00 [23.00, 110.00]	164.00 [61.00, 406.00]	U=1784	0.023
COPD exacerbations (past year)	0.00 [0.00, 1.00]	0.00 [0.00, 1.00]	0.00 [0.00, 1.00]	U=2874	0.507
LOS (days)*	5.00 [4.00, 7.50]	4.00 [3.00, 5.00]	6.00 [4.00, 11.00]	U=1182	7.59×10⁻¹¹
Sex				χ²=0.17	0.681
Male	85 (52.1%)	32 (55.2%)	53 (50.5%)		
Female	78 (47.9%)	26 (44.8%)	52 (49.5%)		
Race				NA	0.7099
Black	155 (95.1%)	54 (93.1%)	101 (96.2%)		
White	6 (3.7%)	3 (5.2%)	3 (2.9%)		
Hispanic	2 (1.2%)	1 (1.7%)	1 (1.0%)		
Insurance type				NA	0.289
Insured	160 (98.2%)	56 (96.6%)	104 (99.0%)		
Uninsured	3 (1.8%)	2 (3.4%)	1 (1.0%)		
Smoking status				NA	0.9371
Never	47 (28.8%)	17 (29.3%)	30 (28.6%)		
Current	82 (50.3%)	29 (35.36%)	53 (64.6%)		
Former	34 (20.9%)	12 (20.7%)	22 (21.0%)		
Illicit drug use (present vs. none)				χ²=0.54	0.461
Present	71 (43.6%)	28 (48.3%)	43 (41.0%)		
None	92 (56.4%)	30 (51.7%)	62 (59.0%)		
Comorbidity: CHF				χ²=4.55	0.0329
Yes	46 (28.2%)	10 (17.2%)	36 (34.3%)		
No	117 (71.8%)	48 (82.8%)	69 (65.7%)		
Comorbidity: CKD				χ²=0.20	0.658
Yes	47 (28.8%)	15 (25.9%)	32 (30.5%)		
No	116 (71.2%)	43 (74.1%)	73 (69.5%)		
Comorbidity: Diabetes				χ²=0.48	0.490
Yes	49 (30.1%)	15 (25.9%)	34 (32.4%)		
No	114 (69.9%)	43 (74.1%)	71 (67.6%)		
Comorbidity: CAD				χ²=1.41	0.235
Yes	20 (12.3%)	10 (17.2%)	10 (9.5%)		
No	143 (87.7%)	48 (82.8%)	95 (90.5%)		
Chest X-ray infiltrate				χ²=1.39	0.238
Yes	56 (34.4%)	16 (27.6%)	40 (38.1%)		
No	107 (65.6%)	42 (72.4%)	65 (61.9%)		
Steroid given*				χ²=0.28	0.597
Yes	124 (76.1%)	46 (79.3%)	78 (74.3%)		
No	39 (23.9%)	12 (20.7%)	27 (25.7%)		
Antibiotics given*				χ²=5.88	0.0153
Yes	103 (63.2%)	29 (50.0%)	74 (70.5%)		
No	60 (36.8%)	29 (50.0%)	31 (29.5%)		

Outcomes and healthcare utilization

Among 163 unique patients (index admissions), ICU admission occurred in 53/163 (32.5%), and NIV was used in 58/163 (35.6%). Endotracheal intubation was less frequent but clinically significant, occurring in 9/163 (5.5%). In-hospital mortality was uncommon, observed in 3/163 (1.8%), consistent with a community hospital mixed-acuity sample in which morbidity and escalation events are more prevalent than death.

Post-discharge utilization remained meaningful, with 30-day readmission documented in 21/163 (12.9%). LOS demonstrated an appreciable utilization burden: using the pre-specified threshold, prolonged LOS (>5 days) occurred in 64/163 (39.3%). When integrated into the pre-specified composite definition, the overall composite adverse outcome occurred in 105/163 (64.4%), indicating that nearly two-thirds of index admissions experienced at least one major adverse utilization or outcome component. Table 2 summarizes these outcomes and utilization measures.

**Table 2:** Outcomes and utilization (index admissions; N=163). ICU: intensive care unit; NIV: noninvasive ventilation; LOS: length of stay.

Outcome	n/N (%)
ICU admission	53/163 (32.5)
NIV used	58/163 (35.6)
Intubation required	9/163 (5.5)
In-hospital mortality	3/163 (1.8)
30-day readmission	21/163 (12.9)
Prolonged LOS (>5 days)	64/163 (39.3)
Composite adverse outcome	105/163 (64.4)

Discharge disposition 

Most patients were discharged home (65%, 106/163), followed by leaving AMA (17.8%, 29/163). Post-acute facility discharges (nursing home, assisted living facility, and skilled nursing facility) and inter-hospital transfers were less frequent; there were three in-hospital deaths. Figure 1 illustrates the discharge disposition.

**Figure 1:**
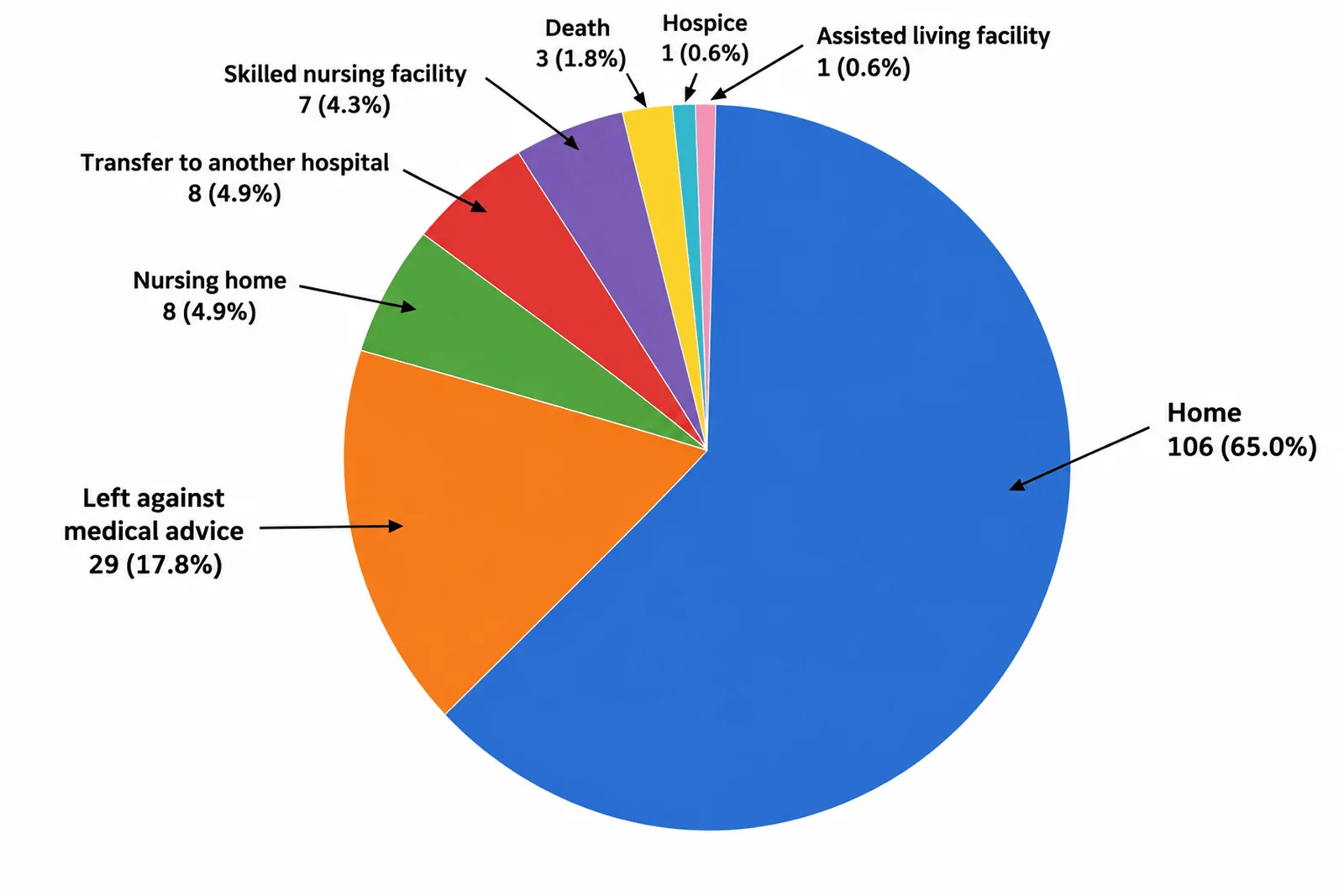
Discharge disposition (index admission; N=163). Index admission defined as the earliest admission date per MRN (repeat admissions excluded). MRN: medical record number.

LOS distribution and escalation-associated LOS

Among 163 index admissions, LOS was right-skewed, with most patients clustering around shorter hospitalizations and a smaller subset experiencing markedly prolonged stays. The median LOS was 5.0 days (IQR 4.0-7.5). Using the pre-specified threshold for prolonged LOS (>5 days), 64 of 163 patients (39.3%) met criteria for prolonged hospitalization. LOS differed substantially according to composite adverse outcome status. Patients without a composite adverse outcome (n=58) had a median LOS of 4.0 days (IQR 3.0-5.0), compared with 6.0 days (IQR 4.0-11.0) among those with a composite adverse outcome (n=105). This difference was statistically significant (Mann-Whitney U=1182; p=7.59×10⁻¹¹), indicating that prolonged hospitalization was an important contributor to the overall composite adverse outcome burden in this cohort. 

LOS also increased in a graded fashion across ventilatory escalation categories. Among patients who required neither NIV nor intubation (n=105), the median LOS was five days. Patients who required NIV without intubation (n=49) had a higher median LOS of six days. The longest hospitalizations occurred among patients who underwent intubation, with or without NIV (n=9), who had a median LOS of 26 days. Figure 2 illustrates the LOS by ventilatory escalation category.

**Figure 2:**
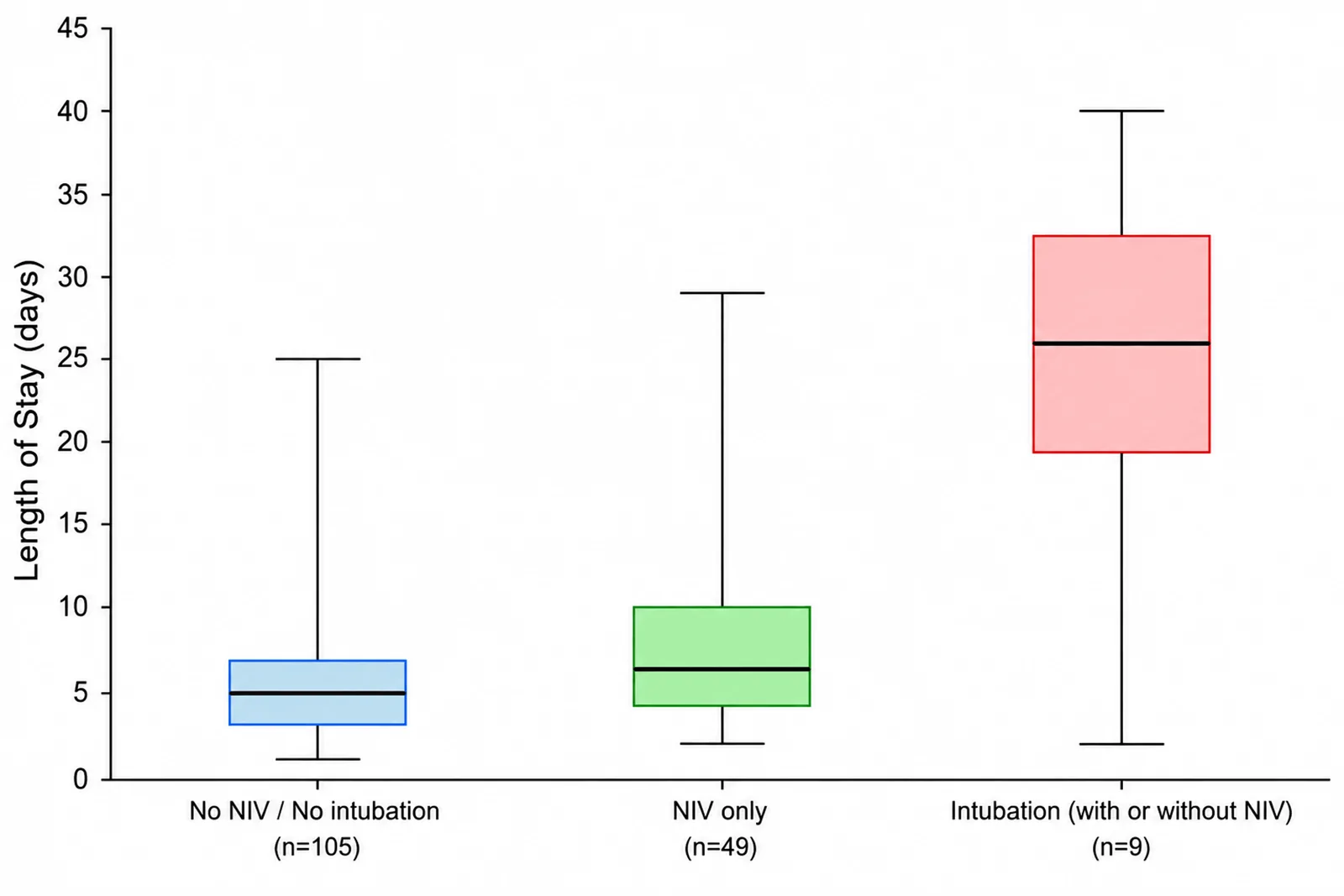
LOS by ventilatory escalation category (index admissions, N=163). NIV: noninvasive ventilation; LOS: length of stay.

Multivariable modeling results

In the multivariable logistic regression model for the composite adverse hospital course outcome during the index admission (Table 3), several independent predictors emerged after adjustment for demographics, comorbidities, admission vital signs and laboratory values, and chest X-ray infiltrates. CHF was independently associated with higher odds of composite adverse outcome, and physiologic severity markers consistent with hypercapnic exacerbation physiology were also significant: higher PaCO₂ (per 10 mmHg increase) was associated with higher odds of composite adverse events. Higher WBC (per 5×10³/µL increase) was also associated with increased odds, consistent with inflammatory burden tracking with more complicated hospital courses. In contrast, several baseline features (e.g., age, sex, smoking status, and most other comorbidities) were not independently associated after adjustment. Table 3 summarizes different predictors for composite adverse hospital course. 

**Table 3:** Multivariable regression model for composite adverse hospital course among unique patients with AECOPD (index admissions only). Effect measure = aOR with 95% CI. Reference categories: Female; not current smoker; no CHF/CKD/diabetes/CAD; no CXR infiltrate. Continuous units: pH per 0.05 decrease; PaCO₂ per 10 mmHg increase; WBC per 5×10³/µL increase; creatinine per 0.5 mg/dL increase; oxygen saturation per 1%. Model fit: AUROC=0.725, AIC=211.3. Predictors are baseline/admission variables. Composite adverse outcome definition: ICU admission, intubation, LOS>5 days, 30-day readmission, or in-hospital mortality. aOR: adjusted odds ratio; ABG: arterial blood gas; AECOPD: acute exacerbation of chronic obstructive pulmonary disease; CHF: congestive heart failure; CKD: chronic kidney disease; CAD: coronary artery disease; WBC: white blood cell; ICU: intensive care unit; AUROC: area under the receiver operating characteristic curve; LOS: length of stay.

Predictor	aOR	95% CI	P-value
Age (per 1 year)	1.00	0.96–1.04	0.985
Male (ref: female)	0.72	0.35–1.51	0.388
Current smoker (ref: not current)	1.09	0.52–2.27	0.814
CHF (ref: no)	3.11	1.15–8.43	0.025
CKD (ref: no)	0.89	0.34–2.33	0.819
Diabetes mellitus (ref: no)	1.04	0.44–2.47	0.920
CAD (ref: no)	0.38	0.12–1.19	0.096
Oxygen saturation (per 1%)	0.99	0.95–1.04	0.757
ABG pH (per 0.05 decrease)	0.61	0.43–0.87	0.006
ABG PaCO₂ (per 10 mmHg increase)	1.94	1.25–3.00	0.003
WBC (per 5 ×10³/µL increase)	1.61	1.01–2.56	0.047
Creatinine (per 0.5 mg/dL increase)	1.13	0.89–1.44	0.317
CXR infiltrate (ref: no)	1.07	0.48–2.39	0.864

Thirty-day readmission occurred in 21/163 (12.9%) unique-patient index admissions. In the parsimonious standard logistic model, CHF (aOR 4.16, 95% CI 1.54-11.20, p=0.00482) and higher creatinine (per 0.5 mg/dL; aOR 1.23, 95% CI 1.05-1.45, p=0.0116) were independently associated with 30-day readmission, while age and AMA discharge were not statistically significant. In the Firth penalized model using the same predictors, effect estimates were attenuated and no predictor met p<0.05 (CHF penalized aOR 1.98, p=0.0833; creatinine penalized aOR 1.25, p=0.106), consistent with reduced small-sample bias and highlighting the exploratory nature of readmission modeling in a low-event setting. Table 4 summarizes parsimonious predictor set for 30-day readmission.

**Table 4:** Parsimonious multivariable models for 30-day readmission among unique AECOPD index admissions (standard logistic and Firth penalized logistic regression). Effect measure=aOR (95% CI). Reference categories: CHF ref=no; AMA ref=no. Creatinine is modeled per 0.5 mg/dL increase. N = 163, readmission events = 21. AECOPD: acute exacerbation of chronic obstructive pulmonary disease; aOR: adjusted odds ratio; CHF: congestive heart failure; AMA: against medical advice.

Predictor	Unit/coding	Standard logistic aOR (95% CI)	P-value	Firth penalized aOR (95% CI)	P-value
Age (years)	Per 1 year	0.99 (0.94, 1.04)	0.717	1.00 (0.96, 1.03)	0.768
CHF (yes vs. no)	1=yes, 0=no	4.16 (1.54, 11.20)	0.00482	1.98 (0.91, 4.29)	0.0833
Creatinine (per 0.5 mg/dL)	Per 0.5 mg/dL	1.23 (1.05, 1.45)	0.0116	1.25 (0.95, 1.65)	0.106
Discharge AMA (yes vs. no)	1=yes, 0=no	2.26 (0.74, 6.92)	0.152	1.34 (0.55, 3.28)	0.522

Negative binomial regression model was used for LOS. In this model, escalation markers were most strongly associated with longer hospitalization. NIV use and especially intubation were independently associated with increased LOS, consistent with these variables capturing higher-acuity respiratory failure and more prolonged recovery needs. Other baseline factors showed weaker or non-significant associations after adjustment, reinforcing that LOS in this cohort is largely shaped by the in-hospital severity trajectory rather than demographics alone. Table 5 summarizes different predictors for LOS. 

**Table 5:** Multivariable negative binomial regression for LOS (index admissions; N=163). Effect measure=aIRR with 95% CI. Reference categories: Female; not current smoker; no CHF/CKD/diabetes/CAD; ICU admission=no; NIV=no; intubation=no. Continuous units: pH per 0.05 decrease; PaCO₂ per 10 mmHg; oxygen saturation per 1%. Model fit: AIC=985.1. Predictors include baseline/admission variables plus in-hospital escalation markers (ICU, NIV, intubation) and are interpreted as severity/utilization associations. LOS: length of stay; aIRR: adjusted incidence rate ratio; CHF: congestive heart failure; CKD: chronic kidney disease; CAD: coronary artery disease; ICU: intensive care unit; NIV: noninvasive ventilation; ABG: arterial blood gas.

Predictor	aIRR	95% CI	P-value
Age (per 1 year)	1.01	0.99–1.03	0.432
Male (ref: female)	1.21	0.86–1.71	0.277
Current smoker (ref: not current)	0.82	0.58–1.17	0.278
CHF (ref: no)	1.10	0.72–1.67	0.656
CKD (ref: no)	1.03	0.70–1.53	0.871
Diabetes mellitus (ref: no)	1.16	0.78–1.74	0.454
CAD (ref: no)	0.62	0.36–1.07	0.086
Oxygen saturation (per 1%)	1.01	0.99–1.03	0.488
ABG pH (per 0.05 decrease)	1.03	0.91–1.16	0.660
ABG PaCO₂ (per 10 mmHg increase)	1.00	0.97–1.03	0.958
ICU admission (ref: no)	1.06	0.69–1.61	0.793
NIV used (ref: no)	1.58	1.03–2.42	0.036
Intubation (ref: no)	2.97	1.36–6.49	0.006

Model discrimination and calibration

For the composite adverse outcome logistic model (index admissions; N=163, events 105/163 [64.4%]), discrimination was acceptable with an AUROC of 0.7192 (95% bootstrap CI 0.6407-0.7977). Decile calibration demonstrated overall agreement between predicted and observed event rates across most risk deciles, with greater deviations in the extreme deciles. For the 30-day readmission parsimonious logistic model (index admissions; N=163, events 21/163 [12.9%]), discrimination was moderate with an AUROC of 0.722 (95% bootstrap CI 0.622-0.862). Decile calibration for readmission showed wider fluctuations in observed event rates across deciles, with the greatest variability in higher-risk strata, consistent with the smaller number of events (Figure 3).

**Figure 3:**
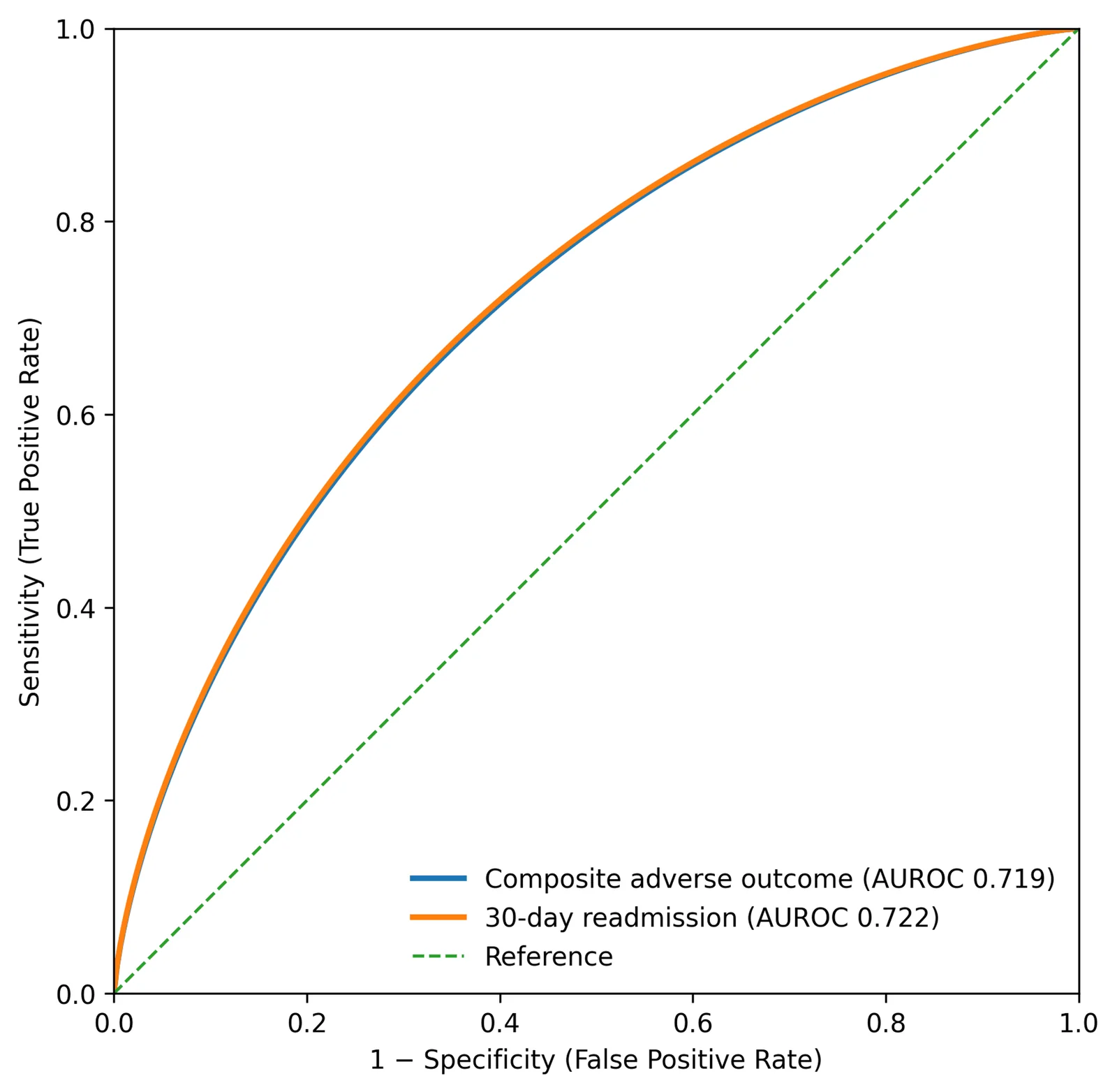
ROC curves for index admission models: composite adverse outcome and parsimonious 30-day readmission. The dashed diagonal line represents no discrimination (AUROC=0.50). Composite adverse outcome model: AUROC 0.7192 (95% bootstrap CI 0.6407-0.7977; N=163, events 105/163). Parsimonious 30-day readmission model (age, CHF, creatinine, AMA discharge): AUROC 0.722 (95% bootstrap CI 0.622-0.862; N=163, events 21/163). AUROC estimates reflect internal/apparent performance in the derivation dataset without external validation. AUROC: area under the receiver operating characteristic curve.

## Discussion

Using index admissions for unique patients to minimize non-independence from repeat hospitalizations, we found that a composite adverse hospital-course endpoint occurred in nearly two-thirds of patients. In multivariable modeling, CHF and marker consistent with hypercapnic respiratory failure severity (higher PaCO₂) were independently associated with the composite adverse endpoint. CHF and renal dysfunction were associated with 30-day readmission in parsimonious modeling. For LOS, escalation markers (NIV and intubation) were associated with longer LOS, consistent with a higher-acuity hospital course.

Hypercapnia and adverse hospital course 

Our finding that a physiologic severity marker related to ventilatory failure (PaCO₂) was associated with adverse composite outcomes is directionally consistent with guideline-based inpatient AECOPD management priorities and prior outcome literature. GOLD emphasizes early severity assessment and escalation to ventilatory support when indicated in hospitalized AECOPD, particularly when hypercapnic respiratory failure is present [3]. The ERS/ATS clinical practice guideline recommends bilevel NIV for AECOPD with acute or acute-on-chronic hypercapnic respiratory failure, supported by evidence that NIV reduces intubation and mortality compared with usual care [4]. The landmark randomized trials by Brochard et al. and Plant et al., and subsequent meta-analysis, established NIV as standard care for appropriately selected patients [6-8]. More recently, HAPPEN demonstrated continued refinement of NIV strategies in hypercapnic AECOPD, underscoring that the hypercapnic phenotype remains central to contemporary inpatient decision-making [9]. While our chart review cannot assess NIV efficacy, our results support the clinical validity of ABG-derived measures as important markers of adverse course, including escalation to invasive support and LOS burden, in real-world community hospital admissions.

Our LOS findings, particularly the marked prolongation among intubated patients and the association of NIV/intubation with longer LOS in regression modeling, align with broader ICU and national observational literature, indicating that escalation pathways in AECOPD identify higher-complexity hospitalizations. Large observational studies have shown that outcomes differ substantially by initial ventilatory strategy and that invasive ventilation or NIV failure is associated with worse outcomes and greater utilization [10,11].

CHF, BNP, and cardiopulmonary multimorbidity

CHF emerged as a consistent predictor across models: it was independently associated with an adverse composite hospital course. This is clinically plausible because dyspnea in AECOPD admissions often reflects overlapping cardiopulmonary pathophysiology, with fluid status, cardiac ischemia, or pulmonary hypertension potentially contributing to severity and prolonged recovery. GOLD highlights multimorbidity, including cardiovascular disease, as integral to COPD care and exacerbation management [3]. BNP was strongly higher in the adverse composite group in univariate comparisons, supporting the concept that myocardial stress/volume-related physiology may track with worse hospital course in this setting.

Readmission: alignment with systematic reviews

Because only 21 30-day readmission events occurred, our primary readmission analysis used a pre-specified parsimonious model (age, CHF, creatinine, AMA discharge) with a Firth penalized logistic sensitivity analysis to mitigate overfitting and small-sample bias. Within this framework, CHF and renal dysfunction (creatinine) emerged as the most consistent readmission correlates, and AMA discharge demonstrated an elevated association in the standard model but with reduced stability under penalization. This pattern is consistent with the broader evidence base that early readmission after AECOPD hospitalization is driven by multimorbidity and physiologic vulnerability as much as pulmonary disease activity alone.

Prior systematic reviews and meta-analyses have repeatedly identified comorbidity burden and markers of clinical complexity as key readmission predictors. In particular, Alqahtani et al. reported that comorbidities (including cardiovascular and renal disease), prior exacerbations/hospitalization history, and longer index LOS were associated with early readmission risk following an AECOPD hospitalization [5]. More recent systematic reviews likewise emphasize that readmission predictors span multiple domains, including comorbid cardiovascular disease, renal impairment, in-hospital course features, and discharge-related factors, supporting the clinical plausibility of our parsimonious readmission predictors in a community hospital setting [13,14]. Taken together, our results align with prior syntheses by highlighting that readmission risk is multifactorial and that readily available inpatient signals (CHF history, creatinine, and discharge context such as AMA) may help identify patients who may benefit from more intensive transitional care planning.

Model discrimination and calibration

Model discrimination and calibration analyses showed that both models provided clinically meaningful internal risk stratification, with more uncertainty for readmission due to fewer events. In the index admission cohort, the composite adverse outcome model demonstrated acceptable discrimination, and the parsimonious 30-day readmission model showed moderate discrimination, indicating that predicted probabilities generally ranked patients with events higher than those without. Decile calibration suggested that predicted risks for the composite endpoint tracked observed event rates reasonably well across most risk strata, whereas calibration for readmission was less stable with greater decile-to-decile variation, which is expected given only 21 readmission events. Importantly, these discrimination and calibration metrics represent internal/apparent performance in the derivation dataset; therefore, they support the models’ potential utility for hypothesis generation.

Strengths

A key strength of this study is the use of an index admission and unique-patient framework, which reduces potential bias associated with repeated hospitalizations and allows for patient-level characterization of risk during an acute exacerbation of COPD. The inclusion of routinely available clinical variables, including comorbidities, ABG parameters, and basic laboratory measurements, enhances the study’s real-world applicability, particularly within community hospital settings where access to advanced biomarkers or specialized testing may be limited. Additionally, the use of a composite morbidity and healthcare utilization endpoint improves statistical tractability in a cohort in which mortality events were relatively uncommon, while capturing clinically meaningful measures of disease severity and healthcare burden.

Limitations

This retrospective, single-center community hospital chart review is limited by potential documentation variability and misclassification inherent to chart abstraction, and the abstraction process (e.g., abstractor training, use of a standardized abstraction form, duplicate abstraction, and inter-rater agreement) may not fully eliminate measurement error across variables and timepoints. Incomplete charts with numerous missing data were excluded from the study. Several operational definitions may vary across institutions, particularly ascertainment of 30-day readmission (often limited to the same health system), ICU admission thresholds, and the timing of “baseline” vitals/labs (e.g., first vs. worst values), which may reduce reproducibility and limit external validity. The modest sample size, low frequency of some outcomes (especially mortality), and heterogeneity of the composite endpoint (with prolonged LOS likely contributing disproportionately and also influenced by nonclinical discharge factors) constrain inference and complicate mechanistic interpretation. Finally, model performance metrics represent internal/apparent performance without external validation, and the readmission model is at risk of overfitting given only 21 events; therefore, findings should be interpreted as associative and hypothesis-generating and require replication in larger, multisite community hospital cohorts.

## Conclusions

In a community hospital setting, adverse hospital course was common among unique-patient index AECOPD admissions, even though in-hospital mortality was low. This supports focusing on morbidity and utilization endpoints (ICU admission, ventilatory escalation, prolonged LOS, and 30-day readmission) as primary outcomes for single-center chart review studies. In multivariable analysis, CHF was a consistent and clinically actionable marker, remaining independently associated with composite adverse hospital course. Physiologic severity markers consistent with hypercapnic exacerbation physiology (higher PaCO₂) and higher inflammatory burden (WBC) were associated with the composite adverse endpoint, reinforcing that early ABG-based severity recognition remains central for inpatient AECOPD risk stratification. For 30-day readmission, a parsimonious modeling strategy supported CHF and renal dysfunction as key correlates, with AMA discharge identifying a potentially actionable transitional care risk factor. These findings support targeted inpatient triage and discharge planning focused on comorbidity burden and early physiologic severity in community hospital AECOPD admissions, while highlighting the need for external validation and prospective evaluation before clinical deployment of prediction tools. 

## References

[1] Wedzicha JA, Seemungal TAR (2007). COPD exacerbations: defining their cause and prevention. Lancet.

[2] Vos T, Lim S, Abbafati C (2020). Global burden of 369 diseases and injuries in 204 countries and territories, 1990-2019: a systematic analysis for the Global Burden of Disease Study 2019. Lancet.

[3] (2025). Global Initiative for Chronic Obstructive Lung Disease (GOLD). Global Strategy for the Diagnosis, Management, and Prevention of COPD: 2025 Report. GOLD.

[4] Rochwerg B, Brochard L, Elliott MW (2017). Official ERS/ATS clinical practice guidelines: noninvasive ventilation for acute respiratory failure. Eur Respir J.

[5] Alqahtani JS, Njoku CM, Bereznicki B (2020). Risk factors for all-cause hospital readmission following exacerbation of COPD: a systematic review and meta-analysis. Eur Respir Rev.

[6] Brochard L, Mancebo J, Wysocki M (1995). Noninvasive ventilation for acute exacerbations of chronic obstructive pulmonary disease. N Engl J Med.

[7] Plant PK, Owen JL, Elliott MW (2000). Early use of non-invasive ventilation for acute exacerbations of COPD on general respiratory wards: a multicentre randomised controlled trial. Lancet.

[8] Lightowler JV, Wedzicha JA, Elliott MW, Ram FS (2003). Non-invasive positive pressure ventilation to treat respiratory failure resulting from exacerbations of chronic obstructive pulmonary disease: Cochrane systematic review and meta-analysis. BMJ.

[9] Luo Z, Li Y, Li W (2024). Effect of high-intensity vs low-intensity noninvasive positive pressure ventilation on the need for endotracheal intubation in patients with an acute exacerbation of chronic obstructive pulmonary disease: the HAPPEN randomized clinical trial. JAMA.

[10] Stefan MS, Nathanson BH, Higgins TL, Steingrub JS, Lagu T, Rothberg MB, Lindenauer PK (2015). Comparative effectiveness of noninvasive and invasive ventilation in critically ill patients with acute exacerbation of chronic obstructive pulmonary disease. Crit Care Med.

[11] Chandra D, Stamm JA, Taylor B (2012). Outcomes of noninvasive ventilation for acute exacerbations of chronic obstructive pulmonary disease in the United States, 1998-2008. Am J Respir Crit Care Med.

[12] Roberts CM, Stone RA, Buckingham RJ, Pursey NA, Lowe D (2011). Acidosis, non-invasive ventilation and mortality in hospitalised COPD exacerbations. Thorax.

[13] Chow R, So OW, Im JHB, Chapman KR, Orchanian-Cheff A, Gershon AS, Wu R (2023). Predictors of readmission, for patients with chronic obstructive pulmonary disease (COPD) - a systematic review. Int J Chron Obstruct Pulmon Dis.

[14] Ruan H, Zhang H, Wang J, Zhao H, Han W, Li J (2023). Readmission rate for acute exacerbation of chronic obstructive pulmonary disease: a systematic review and meta-analysis. Respir Med.

